# Supplementary material for: Cooperation of mitochondrial and ER factors in quality control of tail-anchored proteins
Source: eLife. 2019 Jun 7;8:e45506. doi: 10.7554/eLife.45506 (PMC6586462; doi:10.7554/eLife.45506)
Supplement: Supplementary file 2. [file elife-45506-supp2.docx]

**Supplementary File 2: Array of investigated TA proteins according to Burri and Lithgow (2004)**

| **Standard Name** | **Systematic Name** | **Localization** |
| --- | --- | --- |
| Fmp32 | YFL046W | mitochondrial |
| Fis1 | YIL065C | mitochondrial |
| Tom5 | YPR133W-A | mitochondrial |
| Tom6 | YOR045W | mitochondrial |
| Tom7 | YNL070W | mitochondrial |
| Tom22 | YNL131W | mitochondrial |
| Prm3 | YPL192C | nuclear envelope |
| Kar1 | YNL188W | nuclear envelope |
| Ubc6 | YER100W | ER |
| Scs2 | YER120W | ER |
| Scs22 | YBL091C-A | ER |
| Csm4 | YPL200W | ER |
| Hlj1 | YMR161W | ER |
| Ysy6 | YBR162W-A | ER |
|  | YBL100C | ER |
| Sbh2 |  | ER |
| Dp,1 | YPR183W | ER |
| Sss1 | YDR086C | ER |
| Sbh1 | YER087C-B | ER |
| Pgc1 | YPL206C | ER |
| Pex15 | YOL044W | ER/peroxisome |
| Cyp5 | YNL111C | ER |
| Frt1 | YOR324C | ER |
| Frt2 | YAL028W | ER |
| Far10 | YLR238W | ER |
| Vps64 | YDR200C | ER |
| Sec20 | YDR498C | ER |
| Sec22 | YLR268W | ER |
| Ufe1 | YOR075W | ER |
| Use1 | YGL098W | ER |
| Sed5 | YLR026C | ER-Golgi |
| Gos1 | YHL031C | ER-Golgi |
| Bet1 | YIL004C | ER-Golgi |
| Bos1 | YLR078C | ER-Golgi |
| Tlg1 | YDR468C | Golgi |
| Sft1 | YKL006C-A | Golgi |
| Tlg2 | YOL018C | Golgi |
| Syn8 | YAL014C | Golgi-vacuole |
| Pep12 | YOR036W | Golgi-vacuole |
| Vti1 | YMR197C | Golgi-vacuole |
| Nyv1 | YLR093C | Golgi-vacuole |
| Vam3 | YOR106W | vacuole |
| Snc1 | YAL030W | Plasma membrane-vesicles |
| Snc2 | YOR327C | Plasma membrane-vesicles |
| Sso1 | YPL232W | Plasma membrane |
| Sso2 | YMR183C | Plasma membrane |
|  | YDL012C | Plasma membrane |
| Sps2 | YDR522C | Plasma membrane |
| Phm6 | YDR281C | ? |
|  | YDL241W | ? |
|  | YBL091C-A | ? |
|  | YEL010W | ? |
|  | YEL073C | ? |
|  | YML036W | ? |
