# Supplementary material for: Cooperation of mitochondrial and ER factors in quality control of tail-anchored proteins
Source: eLife. 2019 Jun 7;8:e45506. doi: 10.7554/eLife.45506 (PMC6586462; doi:10.7554/eLife.45506)
Supplement: Supplementary file 3. [file elife-45506-supp3.docx]

**Supplementary File 3: Yeast strains used in this study.**

| **Name** | **Genotype** |
| --- | --- |
| Y8205 | MAT alpha can1Δ::STE2pr-Sp_his5 lyp1Δ::STE3pr-LEU2 his3Δ1 leu2Δ0 ura3Δ0 |
| BY4741 | MAT a his3Δ1 leu2Δ0 met15Δ0 ura3Δ0 |
| YMaM330 | Y8205 leu2Δ::GAL1pr-I-SCEI-natNT2 |
| AK1306 | YMAM330 ho::hisG-URA3-hisG-TEFpr-sfGFP-mCherry-PEX15∆30-CYCt |
| AK1307 | YMAM330 ho::hisG-URA3-hisG-TEFpr-sfGFP-mCherry-TOM5-CYCt |
| YMAM1176 | AK1306 cue1Δ::kanMX6 |
| YMAM1178 | AK1306 get3Δ::kanMX6 |
| YMAM1181 | AK1306 ubc6Δ::kanMX6 |
| YMAM1182 | AK1306 ubc7Δ::kanMX6 |
| YMAM1183 | AK1306 msp1Δ::kanMX6 |
| YVD220 | AK1306 doa10Δ::hphNT1 |
| YVD221 | AK1306 msp1Δ::kanMX6 doa10Δ::hphNT1 |
| YVD222 | AK1306 get3Δ::kanMX6 doa10Δ::hphNT1 |
| AK1305 | YMAM330 ho::hisG-URA3-hisG |
| YVD239 | AK1306 doa10Δ::hphNT1 kanMX6::GAL1pr::MSP1 |
| YVD241 | AK1305 kanMX6::GAL1pr::MSP1 |
| YMAM1205 | MAT alpha, can1Δ::STE3pr-LEU2-GAL1pr-NLS-I-SCEI lyp1Δ his3Δ1 leu2Δ0 ura3Δ0 met15Δ0 |
| YVD244 | YMAM1205 msp1Δ::kanMX6 |
| YVD238 | YMAM1205 doa10Δ::kanMX6 |
| YVD247 | YMAM1205 YKL100C::kanMX6:: YKL100C |
| YVD249 | AK1306 doa10Δ::hphNT1 get2Δ::kanMX6 |
| YVD267 | Y8205 PEX3::mScarlet-i::kanMX6 get3Δ::hphNT1 |
| YVD268 | Y8205 COX4::mScarlet-i::kanMX6 get3Δ::hphNT1 |
| YVD270 | AK1306 msp1Δ::kanMX6 spf1Δ::hphNT1 |
| YVD271 | AK1306 get2Δ::kanMX6 msp1Δ::hphNT1 |
| YVD275 | AK1306 msp1Δ::kanMX6 ubc6Δ::hphNT1 |
| YVD276 | AK1306 get4Δ::kanMX6 |
| YVD277 | AK1306 doa10Δ::hphNT1 get4Δ::kanMX6 |
| YVD279 | AK1306 spf1Δ::hphNT1 |
| YVD280 | AK1306 cue1Δ::kanMX6 hphNT1::GAL1pr::MSP1 |
| YVD281 | AK1306 ubc6Δ::kanMX6 hphNT1::GAL1pr::MSP1 |
| YVD283 | Y8205 ho::hisG-URA3-hisG-TEFpr-sfGFPcp8-PEX15∆30-CYCt COX4::mScarlet-i::kanMX6 |
| YVD284 | Y8205 ho::hisG-URA3-hisG-TEFpr-sfGFPcp8-PEX15∆30-CYCt PEX3::mScarlet-i::kanMX6 |
| YVD292 | Y8205 ho::hisG-URA3-hisG-TEFpr-sfGFPcp8-PEX15Δ30-CYCt COX4::mScarlet-i::kanMX6 msp1Δ::hphNT1 |
| YVD293 | Y8205 ho::hisG-URA3-hisG-TEFpr-sfGFPcp8-PEX15Δ30-CYCt PEX3::mScarlet-i::kanMX6 msp1Δ::hphNT1 |
| YVD294 | Y8205 ho::hisG-URA3-hisG-TEFpr-sfGFPcp8-PEX15Δ30-CYCt PEX3::mScarlet-i::kanMX6 get3Δ::hphNT1 |
| YVD295 | Y8205 ho::hisG-URA3-hisG-TEFpr-sfGFPcp8-PEX15Δ30-CYCt COX4::mScarlet-i::kanMX6 get3Δ::hphNT1 |
| YVD296 | Y8205 ho::hisG-URA3-hisG-TEFpr-sfGFPcp8-PEX15Δ30-CYCt COX4::mScarlet-i::kanMX6 doa10Δ::hphNT1 |
| YVD297 | Y8205 ho::hisG-URA3-hisG-TEFpr-sfGFPcp8-PEX15Δ30-CYCt PEX3::mScarlet-i::kanMX6 doa10Δ::hphNT1 |
| YVD298 | Y8205 ho::hisG-URA3-hisG-TEFpr-sfGFPcp8-PEX15Δ30-CYCt |
| YVD302 | AK1306 get4Δ::kanMX6 hphNT1::GAL1pr::MSP1 |
| YVD303 | AK1306 ubc6Δ::hphNT1 msp1Δ::kanMX6 |
| YVD306 | YMAM1205 doa10Δ::kanMX6 msp1Δ::hphNT1 |
| YVD308 | AK1306 hphNT1::GAL1pr::MSP1 |
| YVD309 | AK1306 get3Δ::kanMX6 hphNT1::GAL1pr::MSP1 |
| YVD311 | Y8205 ho::hisG-URA3-hisG-TEFpr-sfGFPcp8-PEX15∆30-CYCt COX4::mScarlet-i::kanMX6 spf1Δ::hphNT1 |
| YVD312 | Y8205 ho::hisG-URA3-hisG-TEFpr-sfGFPcp8-PEX15∆30-CYCt PEX3::mScarlet-i::kanMX6 spf1Δ::hphNT1 |
| YVD319 | Y8205 ho::hisG-URA3-hisG-TEFpr-sfGFPcp8-PEX15Δ30-CYCt SEC63::mScarlet-i::kanMX6 |
| YVD320 | Y8205 ho::hisG-URA3-hisG-TEFpr-sfGFPcp8-PEX15Δ30-CYCt SEC63::mScarlet-i::kanMX6 msp1Δ::hphNT1 |
| YVD321 | Y8205 ho::hisG-URA3-hisG-TEFpr-sfGFPcp8-PEX15Δ30-CYCt SEC63::mScarlet-i::kanMX6 doa10Δ::hphNT1 |
| YVD322 | Y8205 ho::hisG-URA3-hisG-TEFpr-sfGFPcp8-PEX15Δ30-CYCt SEC63::mScarlet-i-kanMX6 get3Δ::hphNT1 |
| YVD323 | Y8205 ho::hisG-URA3-hisG-TEFpr-sfGFPcp8-PEX15∆30-CYCt SEC63::mScarlet-i::kanMX6 spf1Δ::hphNT1 |
| MKY1585 | MAT a his3200, leu2-3,112, ura3-52, lys2-801 tor1-1, fpr1Δ::klURA |
| YVD376 | MKY1585 pHO::kanMX4-FRB1-sfGFPcp8-Pex15TMD |
| YVD387 | YVD376 p415-TEFpr-HA-FKBP12-Pex15TMD |
| YVD361 | BY4741 leu2Δ0::natNT2-GAL1pr-I-SCEI sfGFP::FMP32 |
| YVD368 | YVD361 msp1Δ::hphNT1 |
| YVD397 | YVD361 doa10Δ::kanMX6 |
| YVD382 | YVD361 msp1Δ::hphNT1 doa10Δ::kanMX6 |
| YVD334 | BY4741 hphΔN-URA3-SpNOP1pr-sfGFP::GEM1 |
| YVD357 | YVD334 msp1Δ::natNT2 |
| YVD404 | YVD334 COX4::mScarlet-i::kanMX6 doa10Δ::natNT2 |
| YVD394 | YVD334 msp1Δ::natNT2 doa10Δ::kanMX6 |
| YVD430 | YVD376 doa10Δ::NatNT2 |
| YVD437 | AK1306 pdr5Δ::kanMX4 |
| YVD440 | AK1306 doa10Δ::hphNT1 pdr5Δ::kanMX4 |
| YVD446 | BY4741 doa10Δ::kanMX4 |
| YVD449 | AK1306 get5Δ::kanMX4 |
| YVD450 | YVD376 p415-TEFpr-HA-FKBP12soluble-CYCt |
| YVD451 | YVD376 doa10Δ::NatNT2 p415-TEFpr-HA-FKBP12soluble-CYCt |
| YVD452 | YVD376 msp1Δ::NatNT2 p415-TEFpr-HA-FKBP12soluble-CYCt |
| YVD459 | BY4741 ho::kanMX4-TEFpr-sfGFP-mCherry-PEX15Δ30-CYCt msp1Δ::kanMX4 |
| YVD460 | BY4741 ho::kanMX4-TEFpr-sfGFP-mCherry-PEX15Δ30-CYCt doa10Δ::kanMX4 |
| YVD462 | BY4741 get3Δ::kanMX4 |
| YVD466 | YVD361 cis1Δ::kanMX4 |
| YVD467 | BY4741 ho::kanMX4-TEFpr-sfGFP-mCherry-Pex15Δ30 doa10Δ::kanMX4 pdr5::hphNT2 |
| YVD472 | YVD376 p415-TEFpr-HA-FKBP12soluble |
| YVD474 | BY4741 MSP1::mNeonGreen leu2Δ0::GAL1pr-NLS-I-SCEI-natNT2 can1Δ::STE2pr-SpHIS5 lyp1Δ::STE3pr-LEU2 |
| YVD475 | YVD474 hphNT1::GAL1pr::MSP1::mNeonGreen |
| YVD490 | YVD474 COX4::mScarlet-i::kanMX6 |
| YVD491 | YVD475 COX4::mScarlet-i::kanMX4 |
| YVD494 | YVD376 p415-TEFpr-HA-mCherry-HA-FKBP12-Pex15TMD-CYCt |
| YVD495 | YVD376 p415-TEFpr-HA-mCherry-HA-FKBP12soluble-CYCt |
| YVD469 | BY4741 pRS426-URA3-Ste6-166::HA |
| YVD470 | BY4741 get3Δ::kanMX4 pRS426-URA-Ste6-166::HA |
| YVD471 | BY4741 doa10Δ::kanMX4 pRS426-URA-Ste6-166::HA |
| YVD478 | BY4741 p415-TEFpr-TAP-Pex15Δ30 pRS413-TEFpr-10xHis-Ubi-CYCt |
| YVD481 | BY4741 msp1Δ::hphNT1 p415-TEFpr-TAP-Pex15Δ30-CYCt pRS413-TEFpr-10xHis-Ubi-CYCt |
| YVD483 | BY4741 doa10Δ::kanMX4 p415-TEFpr-TAP-Pex15Δ30-CYCt pRS413-TEFpr-10xHis-Ubi-CYCt |
| YVD485 | BY4741 p415-TEFpr-TAP-Pex15Δ30-CYCt |
