# Supplementary material for: Cooperation of mitochondrial and ER factors in quality control of tail-anchored proteins
Source: eLife. 2019 Jun 7;8:e45506. doi: 10.7554/eLife.45506 (PMC6586462; doi:10.7554/eLife.45506)
Supplement: Supplementary file 4. [file elife-45506-supp4.docx]

**Supplementary File 4: Plasmids used in this study.**

| **Name** | **Description** | **Source** |
| --- | --- | --- |
| pHO-URA | pHO-URA3 | ATCC# 87805 |
|  | pHO-URA3-TEFpr-sfGFP-mCherry-Tom5-CYCt | V. Okreglak |
|  | pHO-URA3-TEFpr-sfGFP-mCherry-Pex15∆30-CYCt | V. Okreglak |
| p415TEF | p415-TEFpr | V. Okreglak |
|  | p415-TEFpr-sfGFP-mCherry-Tom5 | V. Okreglak |
|  | p415-TEFpr-sfGFP-mCherry-Pex15∆30 | V. Okreglak |
| pHO-Kan | pHO-kanMX4 | ATCC# 87804 |
|  | pHO-kanMX4-TEFpr-tFT-Tom5-CYCt | V. Okreglak |
|  | pHO-kanMX4-TEFpr-tFT-Pex15∆30-CYCt | V. Okreglak |
| pVD20 | pHO-kanMX4-TEFpr-sfGFPcp8-PEX15∆30-CYCt | This study |
| pMaM432 | pFA6a-mCherry-sfGFPcp8-ADHt-kanMX | (Khmelinskii et al. 2016) |
| pMaM500 | pFA6a-mScarlet-i-kanMX6 | M. Knop |
| pFA6a-kanMX4 | pFA6a-kanMX4 | Euroscarf |
| pFA6a-kanMX6 | pFA6a-kanMX6 | (Wach et al., 1994) |
| pFA6a-natNT2 | pFA6a-natNT2 | (Janke et al., 2004) |
| pFA6a-hphNT1 | pFA6a-hphNT1 | (Janke et al., 2004) |
| pMaS120 | GAL1 promoter, no tag, hphNT1 | M. Knop |
| pVD21 | p415-TEFpr-HA-FKBP12-Pex15TMD-CYCt | This study |
| pVD22 | pHO-kanMX4-TEFpr-sfGFPcp8-FRB1-Pex15TMD-CYCt | This study |
| pMaM189-1 | pYM-N-sfGFPΔC-ScURA3-NOP1pr-sfGFP | ([Khmelinskii et al., 2011](#_ENREF_34)) |
| pWO1026 | pRS426-Ste6-166::HA | by D. Wolf |
| pVD27 | p415-TEFpr-HA-FKBP12soluble-CYCt | This study |
| pVD28 | p415-TEFpr-HA-mCherry-HA-FKBP12-Pex15TMD-CYCt | This study |
| pVD29 | p415-TEFpr-HA-mCherry-HA-FKBP12soluble-CYCt | This study |
| pVD30 | p415-TEFpr-TAP-Pex15∆30-CYCt | This study |
| pMAM497-1 | pRS413-HIS-TEFpr-10xHis-Ubi-CYCt | M. Knop |
